# KAnalyze: a fast versatile pipelined K-mer toolkit

**DOI:** 10.1093/bioinformatics/btu152

**Published:** 2014-03-18

**Authors:** Peter Audano, Fredrik Vannberg

**Affiliations:** School of Biology, Georgia Institute of Technology, Atlanta, GA 30332, USA

## Abstract

**Motivation**: Converting nucleotide sequences into short overlapping fragments of uniform length, k-mers, is a common step in many bioinformatics applications. While existing software packages count k-mers, few are optimized for speed, offer an application programming interface (API), a graphical interface or contain features that make it extensible and maintainable. We designed KAnalyze to compete with the fastest k-mer counters, to produce reliable output and to support future development efforts through well-architected, documented and testable code. Currently, KAnalyze can output k-mer counts in a sorted tab-delimited file or stream k-mers as they are read. KAnalyze can process large datasets with 2 GB of memory. This project is implemented in Java 7, and the command line interface (CLI) is designed to integrate into pipelines written in any language.

**Results**: As a k-mer counter, KAnalyze outperforms Jellyfish, DSK and a pipeline built on Perl and Linux utilities. Through extensive unit and system testing, we have verified that KAnalyze produces the correct k-mer counts over multiple datasets and k-mer sizes.

**Availability and implementation**: KAnalyze is available on SourceForge:

https://sourceforge.net/projects/kanalyze/

**Contact:** fredrik.vannberg@biology.gatech.edu

**Supplementary information**: Supplementary data are available at *Bioinformatics* online.

## 1 INTRODUCTION

K-merizing sequence data is a necessary step for many bioinformatics applications. K-mer-based approaches are used to assemble reads, detect repeats, estimate read depth, identify protein binding sites (Newburger and Bulyk, 2009), find mutations in sequencing data (Nordström *et al.*, 2013) and perform a variety of other tasks.

As new applications are created, it is important to have reliable software for generating k-mers. If developers choose to rewrite k-mer code, there is an additional risk of introducing bugs that can affect results. This problem is compounded when algorithms become more complex, such as counting k-mers in large datasets with limited memory. The time required to develop and to test a fast algorithm becomes prohibitive. Existing tools often lack features that make them more available to new applications. Few have an API or document return codes.

We created KAnalyze as a fast reusable k-mer toolkit capable of running on multiple platforms. It is packaged with an API for integration into other programs as well as a CLI for manual execution and scripted pipelines. The count module has a graphical mode for desktop use.

Because it is designed for longevity, the project is organized, documented and tested. The source code includes unit tests to quickly verify accuracy as the code changes. We ran tests on several datasets and compared the results with other k-mer software, including a Perl pipeline we built for verifying results. Throughout the design process, the best practices for scientific computing were observed (Wilson *et al.*, 2014). KAnalyze makes both speed and accuracy available to k-mer applications.

## 2 METHODS

### 2.1 Pipelined components and modules

KAnalyze is organized as a set of modules and components. Modules are pipelines where each step is implemented as a component. Components may be shared among modules. The pipeline runs in parallel with each component passing intermediate results to the next. Input is sent to the first step, and output is written by the last step. Each command line mode executes a single module.

Bounded, synchronized memory queues allow rapid exchange of data through the pipeline. To reduce lock overhead incurred by each queue operation, most components send batches of data elements. By passing intermediate results through memory, disk I/O overhead is avoided.

### 2.2 API and CLI

The API is fully annotated with Javadoc comments for every class, method and field. No method throws any exception without declaring and documenting the conditions under which the exception is thrown. Every constructor and method comment states how null arguments are handled. The web pages generated from the Javadoc comments are available to API developers, and the KAnalyze manual describes how to extend the API.

KAnalyze uses Java's Reflection API to dynamically load some classes. The CLI uses the first command-line argument to find the desired module class. The file reader component uses the file type, such as FASTA or FASTQ, to find the appropriate reader class. As a result, new modules and readers can be added to KAnalyze without modifying existing code.

The CLI is both user-friendly and easily integrated into scripted pipelines. When a program completes, it sends a code back to its caller. Zero is returned to the caller only when the program executes without error. Different types of errors have a specific return codes, and each is documented in the KAnalyze manual. Constants for each return code are defined in the API.

### 2.3 Count module algorithm

The KAnalyze count module counts k-mers in large and small datasets. It works efficiently for datasets too large to fit into memory. Counting takes place in two steps over two components. The split component writes sorted subsets of data to disk, and the merge component accumulates counts from each subset. Split and merge operations can be performed in multiple steps, which allows counting to take place in a distributed environment.

The split component reads k-mers into a memory array until it is full. The array is sorted using Java's Arrays.sort() method, which implements a dual-pivot quicksort algorithm. K-mers are counted by traversing the sorted array. Each k-mer and its count are written to disk, and the location of the output file is sent to the merge component. The memory array is then filled with the next set of k-mers, and a new file of k-mer counts is created. The process repeats until all k-mers have been written.

The merge component reads k-mers and their counts from each file and sums the counts for each k-mer. To avoid loading entire files at once, each file has a small buffer of k-mers. As the files are sorted by k-mer, this module efficiently accumulates k-mer counts and writes a sorted output file.

This modified external merge sort algorithm (Knuth, 1998) efficiently counts all k-mers with limited memory and sorts k-mers as they are processed.

## 3 SOFTWARE TEST RESULTS

We tested KAnalyze performance and accuracy on two public datasets. We obtained human chromosome 1 (Chr1) from UCSC and a randomly chosen dataset from The 1000 Genomes Project Pilot Project 3 (gene region targeted), NA18580. The hg19 Chr1 sequence is a single fully assembled 249 Mb (megabase) sequence, and NA18580 is a set of 1.5 million sequence reads totaling 453 Mb. See Supplementary Section 3.6 for links to these datasets.

We tested KAnalyze 0.9.3, Jellyfish 1.1.10 (Marçais and Kingsford, 2011), DSK 1.5280 (Rizk et al., 2013) and a Perl pipeline we developed for verifying accuracy. These were the latest versions available when testing began. We used Jellyfish hash size 100000000 (10^8^) because it yielded the best performance results. Tests were run on a 12 core machine (2 × Intel Xeon E5-2620) with 32 GB of RAM (DDR3-1600), RAID-6 over SATA drives (3 GB/s, 72K RPM) and CentOS 6.4 (minimal install).

**Fig. 1.**
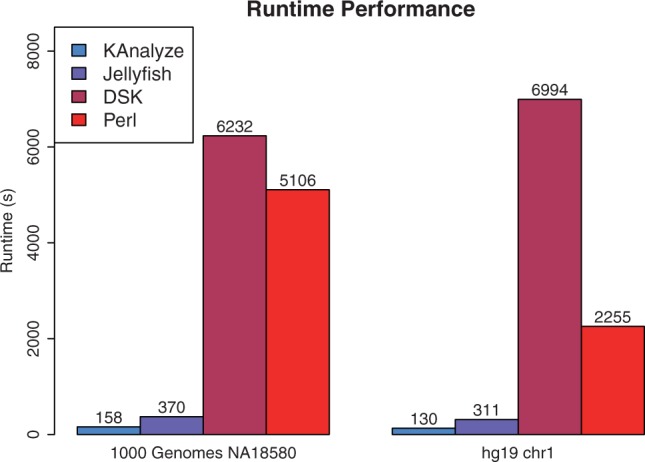
31-mer performance with KAnalyze count, Jellyfish, DSK and a Perl script implementation over two datasets, NA18580 (1000 Genomes) and Chr1 (hg19)

Each pipeline was run in triplicate over both datasets. The run time of each step of each pipeline was recorded with the Linux utility time. The reported time is the average (mean) over all three runs. See Supplementary Section 3 for individual run times. Figure 1 shows the final results.

Memory usage for the NA18580 dataset was determined by recording the maximum RSS (non-swapped physical memory used) in 0.1 s interval with the Linux command ps. For pipelines with multiple steps, we recorded the maximum memory usage of all steps. Actual memory usage was 1.58 GB (KAnalyze count), 2.18 GB (Jellyfish), 0.03 GB (DSK) and 1.96 GB (Perl pipeline). The memory test was done separately from the performance tests.

To test scalability, we obtained HG01889 from the Human Genome Project. This dataset contains 71.95 Gb over 988 million reads. For Jellyfish, we uncompressed the files, which took 1.06 h. In three attempts, we could not get Jellyfish to complete a run on this data in 24 h (see Supplementary Section 3.5). In one attempt, we allowed Jellyfish to use 17 threads, which we determined to be optimal on NA18580. KAnalyze counted k-mers in 14.65 h using 2 GB of memory and default settings in one run. To see how KAnalyze scales in a high-performance setting, we allowed it to use more memory, additional threads and we read directly from the gzipped fastq files. In two tests, KAnalyze counted all k-mers in an average of 3.35 h with 26.01 GB of memory.

For each test, we produced a tab delimited file of k-mers and their counts sorted by k-mer. The KAnalyze sort module (Section 2.3) produces this format. The Perl pipeline produces this format using with Perl scripts with Linux utilities sort and uniq. Jellyfish results were converted from their FASTA representation to a tab-delimited file with a Perl script, and then sorted with Linux sort. DSK produces an unsorted tab-delimited file, which we sorted with Linux sort. The average time to convert and sort results was 901 s for Jellyfish and 507 s for DSK. This time is not shown in Figure 1.

The SHA1 checksum on the sorted output files was recorded. For each dataset, KAnalyze produced results consistent with Jellyfish and the Perl pipeline. DSK k-mer counts did not agree with the other methods (See Supplementary Section 3.4). We obtained the same results running KAnalyze on A Windows computer (Windows 7) and an Apple computer (OS 10.8.5).

## 4 CONCLUSION

KAnalyze offers an extensible API and a complete CLI for k-mer processing tools. These interfaces allow KAnalyze to be integrated directly into Java programs via the API, or into pipelines of any language via the CLI. For desktop users, a graphical interface is included for the count module.

With carefully chosen algorithms and data structures, KAnalyze can perform at a level commensurate with programs compiled to native code. Through extensive testing, we are confident that it produces accurate results.

KAnalyze is designed to survive years of maintenance and feature additions. The source is distributed under the GNU Lesser GPL to restrict its usage as little as possible. We encourage others to contribute to the KAnalyze project.

*Funding*: Georgia Institute of Technology provided financial support through a startup grant to the Vannberg Lab.

*Conflict of Interest*: none declared.

## Supplementary Material

Supplementary Data
