# Supplementary material for: KAnalyze: a fast versatile pipelined K-mer toolkit
Source: Bioinformatics. 2014 Mar 18;30(14):2070–2. doi: 10.1093/bioinformatics/btu152 (PMC4080738; doi:10.1093/bioinformatics/btu152)
Supplement: Supplementary Data [file supp_30_14_2070__index.html]

KAnalyze: A Fast Versatile Pipelined K-mer Toolkit — KAnalyze: a fast versatile pipelined K-mer toolkit — KAnalyze: a fast versatile pipelined K-mer toolkit — Supplementary Data 

# KAnalyze: a fast versatile pipelined K-mer toolkit

## Supplementary Data

files

**Files in this Data Supplement:**

- Supplementary Data - pdf file
