# Supplementary material for: KAnalyze: a fast versatile pipelined K-mer toolkit
Source: Bioinformatics. 2014 Mar 18;30(14):2070–2. doi: 10.1093/bioinformatics/btu152 (PMC4080738; doi:10.1093/bioinformatics/btu152)
Supplement: Supplementary Data [file supp_btu152_KAnalyze_Supplementary.pdf]

KAnalyze: A Fast Versatile Pipelined K-merizer Supplementary Information

Peter Audano and Fredrik Vannberg

1 K-mer Algorithm

The k-mer component converts sequence strings to numeric k-mers. Each nucleotide base is assigned a numeric value (A = 0, C = 1, G = 2, T = 3 and U = 3). The numeric value for a k-mer containing any other letter is undefined. By assigning the same value to T and U, KAnalyze supports DNA and RNA sequences. Any sequence can then be viewed as a base-4 numbering system where the value (V) of the sequence can be assigned as a function of string s with length N. Let s_i be character at string location i such that s_1 is the left-most character in the string, and $v(s_i)$ be the numeric value of the letter s_i as defined above. The k-mer numeric value V is defined as:

$$V = \sum_{i=1}^{N} v(s_i) \cdot 4^{N-i}$$

Because base values are 0 through 3, a numeric k-mer is uniquely represented as a binary number with $2 \cdot k$ bits. This fact along with binary operations in Java can be exploited to create a fast iterative approach to convert sequence strings to numeric k-mers.

After the first k-mer in a sequence string is found, the next k-mer can be derived by removing the leftmost character of the k-mer and appending the next character in the sequence string to the end of the k-mer. This process is repeated until the last character in the sequence string is evaluated. This approach avoids recalculating k-mer information already available in the previous k-mer.

The iterative k-mer process is implemented using Java's primitive long integer data type. Binary shift and bitwise operators offer an efficient method of removing and appending bases. Recall that each base in a k-mer is represented as two bits. Appending the next base is accomplished by using the left-shift operator ($\ll$) to move the existing k-mer left two bits and using the bitwise OR ($|$) to insert the next base at the end of the k-mer. Removing the left-most bits that have fallen off the end of the k-mer is accomplished by applying the binary AND operator (&) with a bitmask containing 1's for the length of the k-mer. See Supplementary Algorithm 1.

Java's primitive long integer is a 64 bit two's complement integer. KAnalyze does not allow negative k-mers, so the left-most bit is not used. Since each base requires 2 bits, the maximum k-mer size in KAnalyze is 31.

Suplementary Algorithm 1: String to numeric k-mer conversion

```
Input: s: A nucleotide sequence
Input: k: Kmer size
Input: mask: Kmer mask
Output: A set of numeric k-mers
M ← ∅                                         ▷ Set of k-mers
kval ← 0                                      ▷ Current k-mer
load ← 1                                      ▷ Number of valid bases in kval
for each character c in s do
    kval ← (kval ≪ 2)
    switch c do
        case A
            ▷ Nothing to add:  A is 0
        case C
            kval | 0x01
        case G
            kval | 0x02
        case T | U
            kval | 0x03
        otherwise
            load ← 0
    if load = ksize then
        M ← M ∪ {kval & mask}
    else
        load ← load + 1
return M
```

2 KAnalyze Count Module

The KAnalyze count module reads sequence files, counts k-mers, and writes a tab-delimited file of k-mers and their counts. The output file is sorted by k-mer. This module is suitable for counting k-mers over small to very large data sets. The amount of memory required is 2GB using default parameters. The required disk space varies with the size and complexity of the data set. For an overview of how this module works, see Section 2.3 of the KAnalyze publication.

The split component uses a fixed-size memory array to accumulate k-mers as they are read. When the memory array is full, a dual-pivot quicksort algorithm distributed in the Java API `Arrays.sort()`[1] sorts the array. The array is then traversed writing all k-mers and their counts to a file on disk. Block I/O, using Java's NIO library, allows files to be written several times faster than Java's streaming I/O libraries. After a file is written to disk, the split component passes the file to the merge component through the pipeline queue. Each of these intermediate files is called a "segment file" in KAnalyze.

As the merge component receives files from the split component, it opens them and prepares to read them. For each file, it creates a small buffer of k-mers and counts to read from. Once the last k-mer has been written to a segment file, the merge component begins to read all segment files from start to end and merge them. Since each segment file is sorted, the output is naturally sorted.

The count pipeline is illustrated in Supplementary Fig. 1

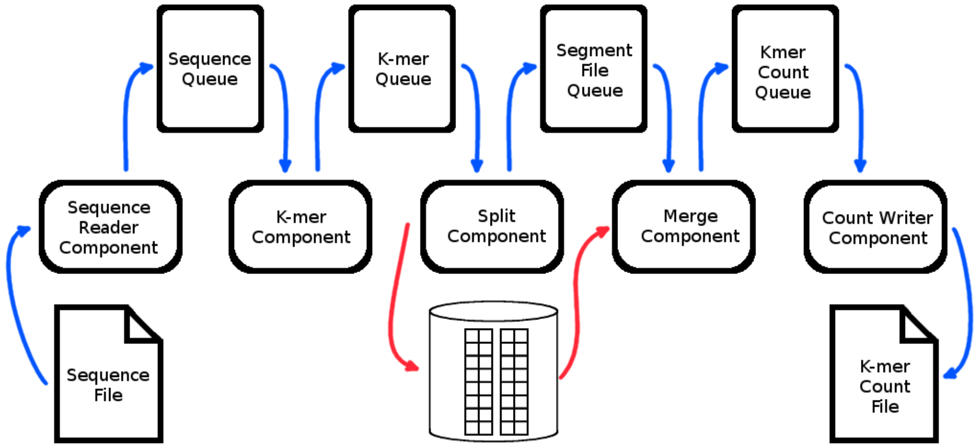

Supplementary Fig. 1: Count pipeline structure. The reader component retrieves sequences from files and passes them to the k-mer component to be k-merized. K-mers are passed to the split component, which fills a memory buffer with k-mers. When split memory buffer is full, k-mers are sorted, counted, and written to segment files. The merge component merges k-mer counts from each segment and passes the k-mers and their counts to the writer component for output. Blue arrows represent passing data through in memory synchronized queues, and red arrows represent passing data to and from disk.

[1]http://docs.oracle.com/javase/7/docs/api/java/util/Arrays.html#sort%28long[]%29

3 Performance Testing

KAnalyze performance was compared against Jellyfish (Marçais and Kingsford, 2011), DSK (Rizk, Lavenier, and Chikhi, 2013), and a pipeline of Perl and Linux tools we built for testing. The Perl/Linux pipeline implements a simple algorithm that was unlikely to contain bugs, which gave us a baseline for comparing results. We include it in performance testing because it represents a simple pipeline that a bioinformatics programmer might create for their own project. See Section 3 of the main publication for details about how tests were executed.

In the main publication, Figure 1 shows the comparative performance of each pipeline. Supplementary Table 1 shows the raw results for the 1000 Genomes data set, and Supplementary Table 4 shows the raw results for hg19 Chr1.

3.1 NA18580

	KAnalyze	Jellyfish	DSK	Perl Pipeline	Jellyfish Post	DSK Post
Run 1	159.80	369.06	5698.36	5100.83	962.02	493.46
Run 2	157.74	377.17	5731.05	5092.60	887.31	490.32
Run 3	157.46	363.92	5744.31	5124.58	853.92	537.34
Mean	158.34	370.05	5724.58	5106.01	901.08	507.04
SD	1.28	6.68	23.65	16.61	55.35	26.29

Suplementary Table 1: 1000 Genomes NA18580 31-mer count performance. Three runtimes for each tool (left of double bar) and the post-processing time to create a sorted tab-delimited file of k-mer counts (right of double bar) for each run are shown. The mean and sample standard deviation are shown for each column.

	Count	Merge	Dump
Run 1	246.21	38.09	84.77
Run 2	245.29	45.36	86.52
Run 3	231.02	38.18	94.72
Mean	240.84	40.54	88.67
SD	8.52	4.17	5.31

Suplementary Table 2: Run times for individual components of Jellyfish on the NA18580 data set. For text output, Jellyfish requires three steps. The first step counts k-mers and writes a set of hash table. The second step merges the hash table files. The last step generates text output. The mean and the standard deviation of each step is shown below the run times. The sum of all steps for a run were added to get the result in Table 1.

	Run	Parse
Run 1	130.58	5567.78
Run 2	131.03	5600.02
Run 3	129.55	5614.77
Mean	130.39	5594.19
SD	0.76	24.03

Suplementary Table 3: Run times for individual components of DSK on the NA18580 data set. DSK requires two steps to create output, Run and Parse. The mean and standard deviation of each step are shown below the run times. The sum of all steps for a run were added to get the result in Table 1.

3.2 Chr1

	KAnalyze	Jellyfish	DSK	Perl Pipeline	Jellyfish Post	DSK Post
Run 1	129.70	311.15	6359.37	2285.71	875.03	581.90
Run 2	128.37	316.22	6336.49	2257.11	923.29	587.83
Run 3	132.69	304.41	6523.09	2222.86	947.19	594.35
Mean	130.25	310.59	6406.32	2255.22	915.17	588.03
SD	2.22	5.93	101.77	31.47	36.76	6.23

Suplementary Table 4: hg19 Chr1 31-mer count performance. Three runtimes for each tool (left of double bar) and the post-processing time to create a sorted tab-delimited file of k-mer counts (right of double bar) for each run are shown. The mean and sample standard deviation are shown for each column.

	Count	Merge	Dump
Run 1	173.04	33.39	104.72
Run 2	185.33	33.34	97.54
Run 3	181.93	33.42	89.06
Mean	180.10	33.38	97.11
SD	6.35	0.04	7.84

Suplementary Table 5: Run times for individual components of Jellyfish on the Chr1 data set. For text output, Jellyfish requires three steps. The first step counts k-mers and writes a set of hash table. The second step merges the hash table files. The last step generates text output. The mean and the standard deviation of each step is shown below the run times. The sum of all steps for a run were added to get the result in Table 4.

	Run	Parse
Run 1	151.58	6207.80
Run 2	150.71	6185.78
Run 3	147.43	6375.65
Mean	149.91	6256.41
SD	2.18	103.85

Suplementary Table 6: Run times for individual components of DSK on the Chr1 data set. DSK requires two steps to create output, Run and Parse. The mean and standard deviation of each step are shown below the run times. The sum of all steps for a run were added to get the result in Table 4.

3.3 Memory Performance

The NA18580 and Chr1 performance tests were repeated measuring memory usage instead of run time. The memory measurement was performance with a script we obtained freely from the internet, `memusg`[2] by Jaeho Shin. This script runs `ps` every 0.1 seconds, records the RSS (non-swapped process memory), and reports the maximum memory usage. For multi-step pipelines, we recorded the maximum memory usage of all steps.

[2] https://gist.github.com/netj/526585

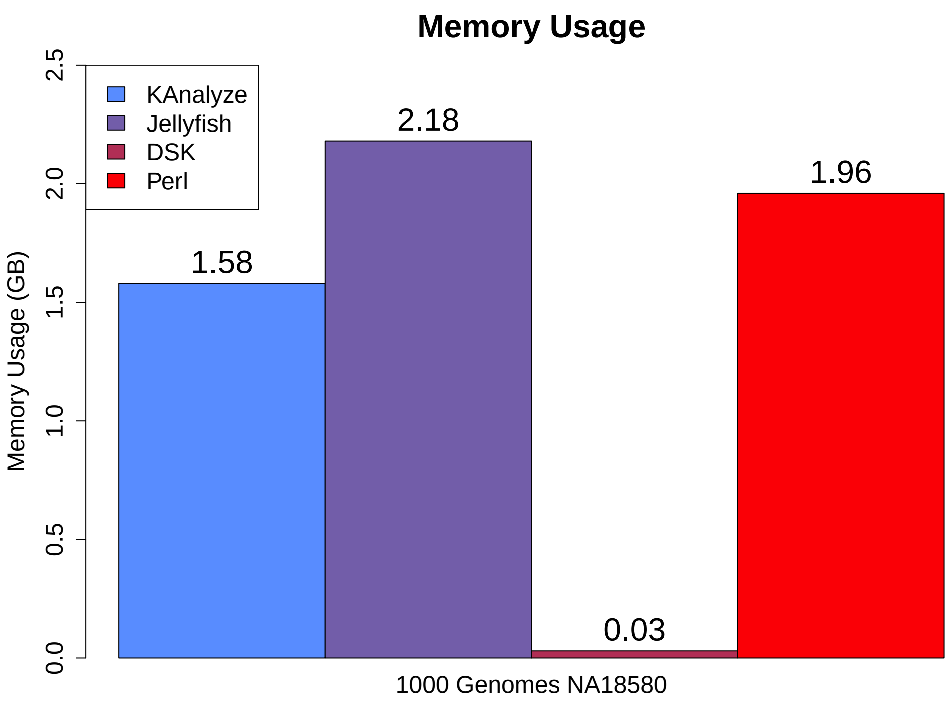

Supplementary Fig. 2: Memory usage for the NA18580 test.

3.4 DSK Performance Test Results

The k-mer counts generated by DSK are not consistent with the counts generated by the other tools we tested for the Chr1 and 1000 Genomes data sets (See 3.6). We found that the number of unique k-mers (k-mers regardless of count) was overstated by 520 (less than 1%) in the Chr1 data, and overstated by 13023703 (37%) in the 1000 Genomes data. The total number of k-mers (by adding all k-mer counts) was understated by 358389 (1%) in the Chr1 data, and understated by 297594 (1%) in the 1000 Genomes data. We did not further investigate these differences.

	DSK	Actual	Difference	Difference %
Total	404,651,774	404,651,774	0	0.00
Unique	183,508,664	195,442,171	11,933,507	6.11

Suplementary Table 7: The difference between DSK and actual k-mers for NA18580. The first row shows differences in the total k-mers. The second row shows the difference in number of unique k-mers found (where each k-mer's count is ignored).

	DSK	Actual	Difference	Difference %
Total	249,250,591	225,279,481	-23,971,110	-10.64
Unique	203,206,154	206,690,014	3,483,860	1.69

Suplementary Table 8: The difference between DSK and actual k-mers for Chr1. The first row shows differences in the total k-mers. The second row shows the difference in number of unique k-mers found (where each k-mer's count is ignored).

For each data set, DSK produced the same results for all thee runs.

3.5 Jellyfish HG01889 Results

We tested Jellyfish on a large data set, HG01889, but the first step (count) did not complete in 3 attempts in under 24 hours. The first two attempts used the same parameters as the performance tests on NA18580 and Chr1. For the third test, we allowed Jellyfish to use 17 threads, which was found to yield the best performance results on NA18580.

	Approx. Wall Time	CPU Time	VIRT	RES	SHR	Files Written
Run 1	25:33:17	25:35:46	52.7g	20g	19g	50
Run 2	24:03:29	24:12:20	53.5g	15g	14g	171
HP Run	24:34:17	54:10:28	23.9g	13g	13g	267

Suplementary Table 9: Before each of the three attempts to run Jellyfish on HG01889 was terminated, a time-stamp and an output from `top` was recorded before terminating the process. The number of hash-table files Jellyfish wrote was also recorded. The time-stamp was recorded within a few minutes of process termination.

3.6 Data Download

The testing data is freely available online.

hg19:
ftp://hgdownload.soe.ucsc.edu/goldenPath/hg19/bigZips/chromFa.tar.gz

1000 Genomes NA18580:
ftp://ftp-trace.ncbi.nih.gov/1000genomes/ftp/data/NA18580/sequence_read/SRR014079.filt.fastq.gz

1000 Genomes HG01889:
ftp://ftp-trace.ncbi.nih.gov/1000genomes/ftp/data/HG01889/sequence_read/

4 Count Performance Bounds

While preparing this publication, we spent a considerable amount of time proving that KAnalyze can process large data sets in a reasonable amount of time. Through developing and testing this software, we gained some valuable information about how it performs and scales. This section discusses the performance boundaries.

In the early stages of KAnalyze, we used Java's standard stream I/O libraries to input and output data. We found that the count split module was spending a great deal of time writing files to disk. In an effort to improve performance, we tried the Java NIO (New I/O) library and found that it wrote files almost 20 times faster than the stream I/O libraries. This took KAnalyze count from being I/O bound to CPU bound.

KAnalyze spends most of its time processing data. Most CPU cycles are spent in the count module phases, split and merge. Split is CPU intensive because it caches k-mers into a large array, sorts them, and writes a segment file. Merge is CPU intensive because it reads from each segment file sequentially and sums k-mer counts from each of them. The performance of both split and merge is directly tied to the size of the array used to cache k-mers in the split phase. A larger array allows split to cache and sort more k-mers per segment file, which results in fewer segment files for the merge component to traverse. The KAnalyze manual discusses performance tuning the count module.

5 Maintenance and Updates

As KAnalyze gains a following, we hope that other programmers will participate with the project by contributing code or documentation. KAnalyze will be a community supported product, and we intend to participate as members of the community. To support this effort, we have designed KAnalyze to be maintainable. We hope that developers will create and share modules they find useful.

We have lowered the barriers to adding features and to fixing bugs as much as possible. We have chosen to license KAnalyze under the GNU LGPL to restrict its usage as little as possible while ensuring updates remain available to everyone. We have chosen SourceForge to distribute the code and the KAnalyze packages because we can allow others to send us updates. If you are interested in becoming a contributor, please visit the KAnalyze project on SourceForge (https://sourceforge.net/projects/kanalyze/).

References

Marçais, G. and Kingsford, C. (2011). A fast, lock-free approach for efficient parallel counting of occurrences of k-mers. *Bioinformatics (Oxford, England)*, **27**(6), 764–70.

Rizk, G., Lavenier, D., and Chikhi, R. (2013). DSK: k-mer counting with very low memory usage. *Bioinformatics (Oxford, England)*, **29**(5), 652–3.
